# Correlation between Heart rate recovery and Left Atrial phasic functions evaluated by 2D speckle-tracking Echocardiography after Acute Myocardial infarction

**DOI:** 10.1186/s12872-023-03194-y

**Published:** 2023-03-29

**Authors:** Behruz Mashayekhi, Reza Mohseni-Badalabadi, Ali Hosseinsabet, Tahereh Ahmadian

**Affiliations:** 1 Cardiology Department, Tehran Heart Center, Tehran University of Medical Sciences, Karegar Shomali Street, Tehran, I.R. of Iran; 2 Research Department, Tehran Heart Center, Tehran University of Medical Sciences, Tehran, I.R. of Iran

**Keywords:** Myocardial infarction, Left atrium, Two-dimensional speckle-tracking echocardiography, Heart rate recovery, Exercise test

## Abstract

**Background:**

Heart rate recovery (HRR) in the exercise test is the index of cardiac autonomic system function and sympathovagal balance impaired in patients with myocardial infarction (MI). An instance is left atrial (LA) phasic function, which is impaired in such patients. In this study, we investigated the role of HRR in predicting LA phasic functions in patients with MI.

**Methods:**

The present study recruited 144 consecutive patients with ST-elevation MI. A symptom-limited exercise test was performed about 5 weeks after MI, with echocardiography conducted just before the exercise test. The patients were divided into abnormal and normal HRR at 60 s (HRR60) and again into abnormal and normal HRR at 120 s (HRR120) after the exercise test. LA phasic functions, evaluated by 2D speckle-tracking echocardiography, were compared between the 2 groups.

**Results:**

Patients with abnormal HRR120 had lower LA strain values and strain rates during the reservoir, conduit, and contraction phases, while those with abnormal HRR60 had lower LA strain values and strain rates during the reservoir and conduit phases. The differences were lost after adjustments for possible confounders, except for LA strain and strain rate during the conduit phase, in patients with abnormal HRR120.

**Conclusions:**

Abnormal HRR120 in the exercise test can independently predict decreased LA conduit function in patients with ST-elevation MI.

## Introduction

Myocardial infarction (MI) affects approximately 3% of the population over 20 years of age in the United States, where every 40 s, 1 MI occurs. [1] The left atrial (LA) walls are rich in terms of the presence of sympathetic and parasympathetic neurons. MI can lead to an imbalance in sympathovagal output, contributing to post-MI ventricular and atrial arrhythmias. [2, 3] In addition, LA phasic functions are affected after MI [4] and are predictive of adverse clinical events. [5–7] LA phasic functions are associated with exercise capacity in various conditions ranging from normal conditions to various types of heart failure, including post-MI scenarios. [8–12]

Heart rate recovery (HRR) in the exercise test is defined as the difference between the maximal heart rate at the exercise time and the heart rate at a defined recovery time. HRR is the index of cardiac autonomic system function and sympathovagal balance. [13] It is a prognostic factor in the general population and patients with coronary artery disease. [14–16] Heart rate variability (HRV) is another marker of cardiac autonomic system function and is evaluated by electrocardiography monitoring. Although the association between HRR and HRV has been presented in some studies, this association is not strong [17, 18], which may suggest different aspects of cardiac autonomic system function assessed by these markers. [19] HRV is associated with LA phasic functions in patients with hypertension and diabetes. [20, 21] Nonetheless, the data regarding the association between HRR and LA phasic functions are scarce. [22] In comparison with HRV, HRR is rapidly obtained from the exercise test. Additionally, the exercise test alone provides much information regarding the cardiovascular system.

Two-dimensional speckle-tracking echocardiography (2DSTE) is widely used to evaluate LA phasic functions. It assesses the deformation of the LA myocardium during the cardiac cycle, especially in the longitudinal direction, enabling the evaluation of the 3 LA phasic functions: reservoir, conduit, and contraction. In brief, the blood is reserved in the LA during systole; then, it is directed into the left ventricle (LV) at early diastole before it is pushed into the LV by LA contraction at late diastole. [23]

We hypothesized that cardiac autonomic system function was associated with LA phasic functions in patients with recent acute MI and aimed to evaluate the association between LA phasic functions, assessed by 2DSTE, and HRR in symptom-limited exercise tests in patients with a history of recent acute MI. The findings should further contribute to our understanding of the interaction between cardiac autonomic system function and cardiac mechanics.

## Methods

### Study population

From September 2020 through July 2021, the present study included patients who underwent successful primary percutaneous coronary intervention due to acute MI (thrombolysis in MI grade I or 0) in our hospital. Acute MI was defined in accordance with the fourth universal definition for ST-elevation MI. [24] The exclusion criteria were composed of inability to do the exercise test according to the patient’s expression, neglected MI, atrial fibrillation (AF) rhythm, left bundle branch block, moderate and more-than-moderate valvular regurgitation, any degree of valvular stenosis, a history of previous MI, percutaneous coronary intervention, cardiac surgery, pacemaker implantation, congenital heart disease, cancer, autoimmune disease, hepatic failure, creatinine > 1.5 mg/dL, uncontrolled thyroid disease, cardiomyopathies, and poor echocardiography windows. Finally, 144 consecutive patients were included in our study. Overnight fasting venous blood was drained in the morning after admission for biochemistry and blood cell count. The patients were treated according to validated recommendations. [25–27] Hypertension was defined as antihypertensive drug consumption or a history of blood pressure > 140/90 mm Hg in 2 isolated measurements. Diabetes was defined as the consumption of an antidiabetic drug or insulin, hemoglobin A1c levels > 6.4%, or a history of fasting blood glucose levels ≥ 126 mg/dL in 2 separate samples.

The post-discharge echocardiographic examination and symptom-limited exercise test, scheduled approximately 5 weeks after discharge, were compatible with the first post-discharge outpatient visits in our hospital. Echocardiography was followed instantly by the exercise test. The drugs used by the patients at echocardiography time were recorded. The research proposal was approved by our hospital’s review board, and written informed consent was obtained from all the patients.

### Standard echocardiography

A cardiologist with nearly a decade of experience in advanced echocardiography performed all standard and 2DSTE examinations. Echocardiography was conducted with the patients in the left lateral decubitus position. One-lead electrocardiography monitoring was done continuously. A commercial echocardiography machine (Philips, Affinity 70 C, Andover, MA, USA) with an S5-1 probe was used. LV end-systolic and end-diastolic volumes were measured in the apical 2 and 4-chamber views according to the modified Simpson method; subsequently, the left ventricular ejection fraction (LVEF) was calculated. Pulsed-wave Doppler was applied to record the mitral inflow wave and the pulmonary vein flow. The peak of the mitral early and late diastolic waves (E and A, respectively), the deceleration time of the E wave, and the peak of the pulmonary vein flow in systole and diastole (S and D, respectively) were measured in 3 consecutive cardiac cycles, and their average was presented. Pulsed-wave tissue Doppler was utilized to record myocardial velocities at the septal and lateral mitral annuli. The peak velocities in systole, early diastole, and late diastole (s′, e′, and a′, respectively) in 3 consecutive cardiac cycles were measured, and the average velocity of the septal and lateral mitral annuli was demonstrated. Next, the E/averaged e′ ratio was computed. All the measurements, including diastolic dysfunction severity, [28] were done following the recommendations of the American Society of Echocardiography. [29, 30]

### 2DSTE

For 2DSTE on the LA, 3 cardiac cycles in the 2 and 4-chamber views in the expiratory phase were obtained, with maximal efforts applied to avoid the foreshortening of the LA and the inclusion of the LA appendage and the pulmonary vein orifice. The echocardiography movies had a rate of 48 ± 6 frames per second.

**Fig. 1:**
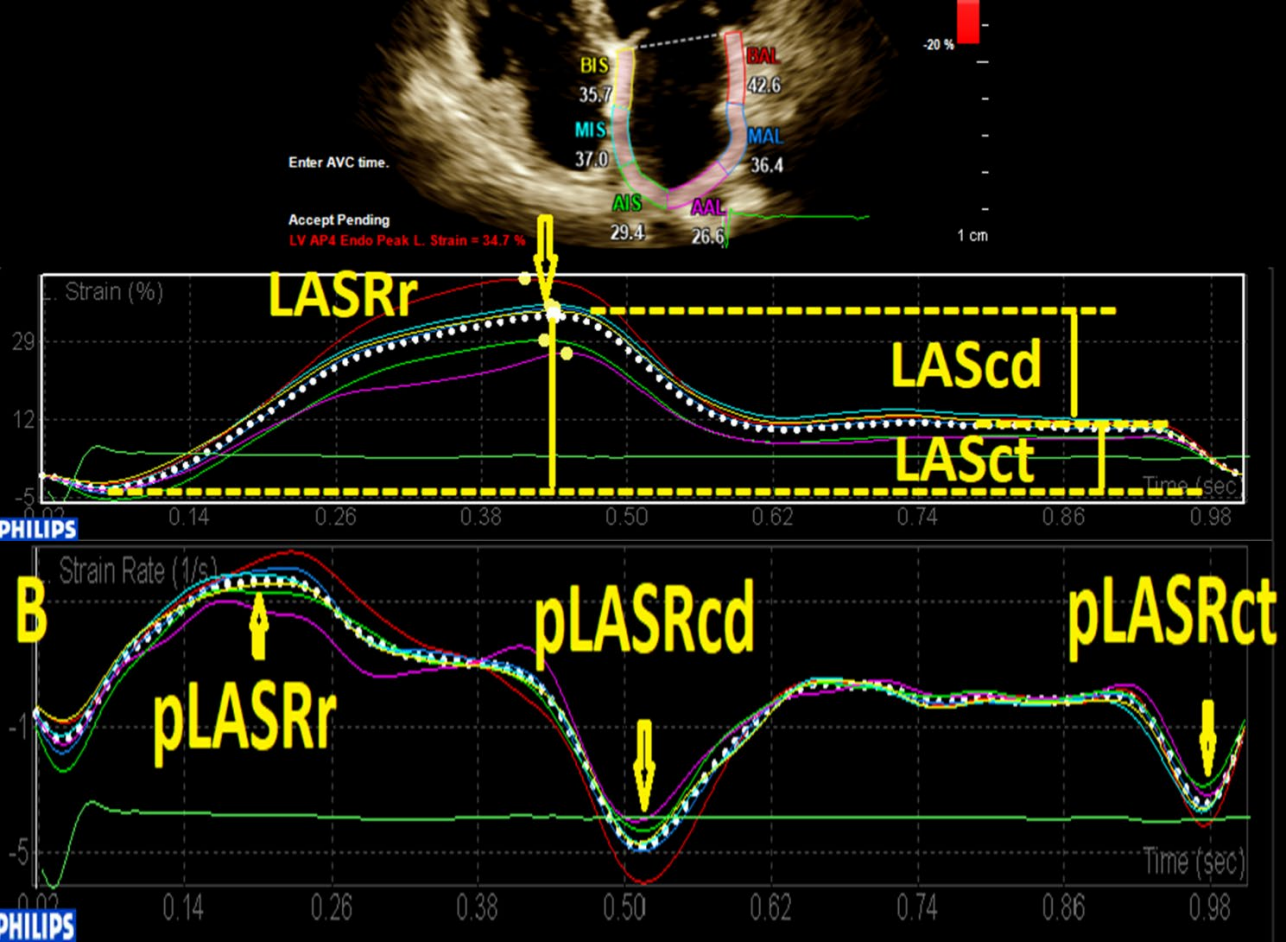
The image illustrates the 2D speckle-tracking echocardiography of the left atrium in the 4-chamber apical view. (A) Strain curves (B) Strain rate curves LAScd, Left atrial longitudinal strain during the conduit phase; LASct, Left atrial longitudinal strain during the contraction phase; LASr, Left atrial longitudinal strain during the reservoir phase; pLASRcd, Peak left atrial longitudinal strain rate during the conduit phase; pLASRct, Peak left atrial longitudinal strain rate during the contraction phase; pLASRr, Peak left atrial longitudinal strain rate during the reservoir phase

The aCMQ option in the QLAB 13.0 package was employed to evaluate longitudinal deformation markers in the LA myocardium. First, the endocardial and epicardial borders were traced as 5 mm segments, and the endocardial layer strain and the strain curve were selected for visualization. Next, at end-diastole, via the 3-click method, the 2 sides of the mitral annulus and the center of the LA roof were pointed. Afterward, the endocardial and epicardial borders of the LA were automatically traced by software and divided into 6 segments. The operator manually adjusted the traced border with the actual endocardial and epicardial borders if required. In the next step, with the aid of the compute option, the strain and strain rate curves were illustrated while the peak of the R wave was set as level 0. If 1 of the segment curves was noisy, the aforementioned steps were repeated. The global strain curve was composed of 3 parts: 1 positive peak at systole, 1 plateau at early diastole, and 1 negative peak at late diastole. The difference between a positive peak and a negative peak was presented as LASr, the difference between a positive peak and a plateau as LAScd, and the difference between a plateau and a negative peak as LASct. The strain rate curve had 1 positive peak at systole and 2 negative peaks at early and late diastole, and it was presented as pLASRr, pLASRcd, and pLASRct, respectively (Fig. 1). These parameters were measured in 3 cardiac cycles, and their mean value was presented. LASr and pLASRr were the indices of the LA reservoir function, LAScd and pLASRcd were the indices of the LA conduit function, and LAScd and pLASRcd were the indices of the LA contraction function. LA 2DSTE was done following the recommendations of the American Society of Echocardiography. [31]

The aCMQ option provides curve volume changes during the cardiac cycle. Hence, we measured maximal, minimal, and pre-P LA volumes before computing the volumetric parameters of LA functions. The left atrial total emptying volume (LATEV) was considered the difference between maximal and minimal LA volumes. The left atrial passive emptying volume (LAPEV) was considered the difference between maximal and pre-P LA volumes. The left atrial active emptying volume (LAAEV) was considered the difference between pre-P and minimal LA volumes.

LATEV multiplied by 100 and divided by LA maximal volume provides the LA total emptying fraction, and LATEV multiplied by 100 and divided by LA minimal volume yields the expansion index as 2 markers of the LA reservoir function.

LAPEV multiplied by 100 and divided by maximal LA volume provides the LA passive emptying fraction, and LAPEV divided by LATEV yields the passive emptying percentage of total emptying as 2 markers of the LA conduit function.

LAAEV divided by pre-P LA volume yields the LA active emptying fraction, and LAAEV divided by LATEV provides the booster active emptying percentage of total emptying as 2 markers of the LA contraction function.

The inter and intraobserver variabilities of the 2DSTE-derived markers of LA phasic functions were calculated 3 months after the termination of the analysis. Twenty-four (17%) patients were randomly selected for the analysis of inter and intraobserver variabilities. Another cardiologist, highly experienced in advanced echocardiography, and the previously mentioned cardiologist evaluated the interobserver variability independently.

### The exercise test

The exercise test was done under an experienced nurse’s observation and in accordance with the Bruce protocol, including a 2-stage cooldown, with a commercial setting (Phillips, STi80 Stress Testing System, Andover, USA). The patients were asked to do the exercise until the completion of the protocol or the appearance of symptoms such as dyspnea, dizziness, chest pain, and ST depression > 1 mm. Continuous 12-lead electrocardiography monitoring was conducted during the exercise test, in conjunction with blood pressure monitoring at the end of each stage. The exercise duration, the achieved metabolic equivalent, the maximal heart rate at the exercise time, and the heart rate at 60 and 120 s were recorded. Next, HRR at 60 s (HRR60) and HRR at 120 s (HRR120) were computed. HRR60 values ≤ 12 bpm and HRR values < 22 bpm were considered abnormal. [32] Forty-five patients had abnormal HRR60, 35 patients had abnormal HRR120, 99 patients had normal HRR60, and 109 patients had normal HRR120.

### Statistical analysis

Categorical data were shown as frequencies and percentages and compared using the χ^2^ test or the Fisher exact test, whichever was appropriate. Normally distributed continuous data were demonstrated as mean values and standard deviations and compared using the independent Student *t* test; otherwise, they were presented as median values and interquartile ranges (25th–75th) and compared using the Mann–Whitney *U* test. Variables that were different between the 2 groups (*P* < 0.05) were considered potential confounders and entered into a multivariable regression analysis if they were physiologically supported and compatible with the assumptions of the multivariable regression analysis. If the dependent variables were not normally distributed, they were first transformed logarithmically; then, the logarithm of that variable was included in the multivariable regression analysis. Inter and intraobserver variabilities were evaluated using intraclass correlation coefficients. A *P* value < 0.05 was considered statistically significant, and all the statistical analyses were done using IBM SPSS Statistics for Windows, version 24 (Armonk, NY: IBM Corp).

## Results

First, the characteristics, laboratory, and echocardiography data were compared between patients with HRR60 ≤ 12 bpm and those with HRR60 > 12 bpm (abnormal vs. normal). Then, these data were compared between patients with HHR120 bpm < 22 bpm and HRR120 ≥ 22 bpm (abnormal vs. normal). The results of these comparisons are presented in Tables 1, 2, 3 and 4. All the patients used antiplatelet agents.

**Table 1:** Demographic, clinical, and laboratory data of the studied groups

Variables	HRR60 ≤ 12 bpm (N = 45)	HRR60 > 12 bpm (N = 99)	*P* value	HRR120 < 22 bpm (N = 35)	HRR120 ≥ 22 bpm (N = 109)	*P* value
Age (y)	58.8 ± 9.4	53.2 ± 8.9	0.001	60.6 ± 8.5	53.1 ± 8.9	< 0.001
Male sex (%)	36 (80)	90 (91)	0.067	30 (86)	96 (88)	0.770
Body mass index (kg/m^2^)	27.8 ± 4.4	27.5 ± 3.8	0.724	27.7 ± 4.1	27.6 ± 4.0	0.819
Body surface area (m^2^)	1.8 ± 0.2	1.9 ± 0.2	0.052	1.8 ± 0.2	1.9 ± 0.2	0.054
Obesity (%)	12 (27)	22 (22)	0.561	10 (29)	24 (22)	0.427
Hypertension (%)	21 (47)	26 (26)	0.016	16 (46)	31 (28)	0.058
Diabetes (%)	23 (51)	26 (26)	0.004	18 (51)	31 (28)	0.013
Cigarette smoking (%)	21 (47)	56 (57)	0.270	16 (46)	61 (56)	0.290
Family history of CAD (%)	14 (31)	32 (32)	0.885	12 (34)	34 (31)	0.733
History of ACEI/ARB usage (%)	41 (91)	93 (94)	0.504	30 (86)	104 (95)	0.063
History of β-blocker usage (%)	41 (91)	95 (96)	0.257	32 (91)	104 (95)	0.403
History of calcium channel blocker usage (%)	4 (9)	7 (7)	0.740	3 (9)	8 (7)	0.729
History of nitrate usage (%)	23 (51)	43 (43)	0.391	16 (46)	50 (46)	0.987
History of statin usage (%)	42 (93)	95 (96)	0.678	32 (91)	105 (96)	0.361
History of diuretic usage (%)	8 (18)	19 (19)	0.840	8 (23)	19 (17)	0.474
History of oral antidiabetic agent usage (%)	21 (47)	23 (23)	0.005	17 (49)	27 (25)	0.008
History of insulin usage (%)	1 (2)	2 (1)	> 0.999	0 (0)	3 (3)	> 0.999
LAD culprit lesion (%)	30 (67)	53 (54)	0.139	22 (63)	61 (56)	0.473
LCX culprit lesion (%)	5 (11)	13 (13)	0.734	2 (6)	16 (15)	0.242
RCA culprit lesion (%)	10 (22)	33 (33)	0.177	11 (31)	32 (29)	0.816
Single-vessel disease (%)	19 (42)	52 (53)	0.369	15 (43)	57 (52)	0.331
Two-vessel disease (%)	16 (36)	25 (25)	0.204	15 (43)	26 (24)	0.030
Three-vessel disease (%)	9 (20)	22 (22)	0.764	5 (14)	26 (24)	0.231
FBS (mg/dL)	138 (109–220)	113 (98–142)	0.007	134 (107–183)	114 (99–147)	0.006
Creatinine (mg/dL)	1.0 ± 0.2	1.0 ± 0.2	0.584	1.1 ± 0.1	1.0 ± 0.2	0.401
Hemoglobin (g/dL)	14.7 ± 1.7	15.4 ± 1.4	0.021	14.9 ± 1.6	15.3 ± 1.5	0.192
Triglyceride (mg/dL)	130 (100–173)	131 (102–184)	0.602	114 (98–173)	134 (107–183)	0.271
Cholesterol (mg/dL)	163 ± 35	169 ± 46	0.442	164± 34	168 ± 45	0.647
HDL (mg/dL)	43 ± 10	39 ± 9	0.019	39 ± 9	44 ± 9	0.014
LDL (mg/dL)	103 ± 25	108 ± 32	0.395	104 ± 25	107 ± 31	0.558

ACEI/ARB, Angiotensin-converting enzyme inhibitor/angiotensin-receptor blocker; CAD, Coronary artery disease; FBS, Fasting blood sugar; HDL, High-density lipoprotein; LAD, Left anterior descending artery; LCX, Left circumflex artery; LDL, Low-density lipoprotein; RCA, Right coronary artery

**Table 2:** Standard echocardiography data and volumetric parameters of the left atrium in the studied groups

Variables	HRR60 ≤ 12 bpm (N = 45)	HRR60 > 12 bpm (N = 99)	*P* value	HRR120 < 22 bpm (N = 35)	HRR120 ≥ 22 bpm (N = 109)	*P* value
LVEDV index (mL/m^2^)	53 ± 11	52 ± 12	0.845	55 ± 13	52 ± 12	0.232
LVESV index (mL/m^2^)	26 ± 9	23 ± 8	0.138	24 (19–34)	22 (19–26)	0.063
LVEF (%)	47 ± 10	51 ± 9	0.018	47 ± 11	51 ± 9	0.041
E (cm/s)	68 ± 22	61 ± 14	0.056	65 ± 21	62 ± 16	0.415
A (cm/s)	74 ± 21	62 ± 16	0.002	75 ± 22	63 ± 16	0.001
E/A ratio	1.0 ± 0.4	1.0 ± 0.4	0.275	0.9 (0.6–1.1)	0.9 (0.8–1.3)	0.039
DT (ms)	216 ± 56	219 ± 54	0.800	224 ± 61	216 ± 52	0.433
S (cm/s)	56 ± 12	52 ± 10	0.086	55 ± 12	53 ± 10	0.271
D (cm/s)	43 ± 16	40 ± 12	0.260	41 ± 12	41 ± 12	0.774
S/D ratio	1.4 ± 0.4	1.4 ± 0.4	0.713	1.4 ± 0.4	1.4 ± 0.4	0.301
Mean s´(cm/s)	7.4 ± 1.6	8.5 ± 1.7	< 0.001	7.3 ±1.6	8.5 ± 1.7	< 0.001
Mean e´(cm/s)	7.4 ± 1.7	8.6 ± 1.8	< 0.001	7.0 ± 1.7	8.5 ± 1.8	< 0.001
Mean a´(cm/s)	9.1 ± 1.7	9.4 ± 1.5	0.397	9.0 ± 1.9	9.4 ± 1.5	0.235
Average e´/a´	0.8 ± 0.2	0.9 ± 0.2	0.014	0.8 ± 0.2	0.9 ± 0.2	0.007
E/(average e´)	9.0 (6.6 - 11.6)	7.0 (5.9–8.3)	< 0.001	9.0 (6.5–12.0)	7.0 (6.0–8.0)	0.004
Systolic pulmonary arterial pressure (mm Hg)*	29 ± 6	27 ± 7	0.166	29 ± 6	27 ± 7	0.139
Diastolic dysfunction grades II and III**	7 (16)	4 (4)	0.022	5 (14)	6 (6)	0.095
LA enlargement [LA maximal volume index > 35 (mL/m^2^)]	15 (33)	19 (19)	0.064	15 (43)	19 (17)	0.002
Maximal LA volume index (mL/m^2^)	31.2 ± 8.3	28.7 ± 7.2	0.070	33.3 ± 7.6	28.3 ± 7.2	< 0.001
Minimal LA volume (mL/m^2^)	11.3 (9.4–16.2)	10.8 (8.0-12.9)	0.034	14.5 (10.5–17.3)	10.3 (7.9–12.6)	< 0.001
Pre-A LA volume (mL/m^2^)	23.4 ± 7.2	21.0 ± 6.1	0.044	25.8 ± 6.2	20.4 ± 6.1	< 0.001
LA total emptying fraction (%)	60 ± 8	62 ± 8	0.084	57 ± 7	63 ± 8	< 0.001
Expansion index (%)	157 (118–197)	164 (138–204)	0.096	130 (116–163)	171 (141–210)	< 0.001
LA passive emptying fraction (%)	26 ± 8	27 ± 8	0.241	23 ± 6	28 ± 6	< 0.001
Passive emptying percentage of total emptying (%)	43 ± 11	44 ± 10	0.617	40 ± 10	45 ± 10	0.009
LA active emptying fraction (%)	46 ± 9	48 ± 8	0.141	44 ± 8	48 ± 8	0.016
Booster active emptying percentage of total emptying (%)	57 ± 11	56 ± 11	0.617	60 ± 10	55 ± 10	0.009

**Systolic pulmonary artery pressure was measurable in 25 patients with HRR ≤ 12 bpm, 56 patients with HRR > 12 bpm, 25 patients with HRR < 22 bpm, and 62 patients with HRR ≥ 22 bpm.

**One patient was in the indeterminate category

HRR60; Heart rate recovery at 60 s after exercise termination (Maximal heart rate – Heart rate at 60 s after exercise termination), HRR120; Heart rate recovery at 120 s after exercise termination (Maximal heart rate – Heart rate at 120 s after exercise termination);

DT, Deceleration time; LA, Left atrium; LV, Left ventricle; LVEDV, Left ventricular end-diastolic volume; LVEF, Left ventricular ejection fraction; LVESV, Left ventricular end-systolic volume

**Table 3:** Exercise test data in the studied groups

Variables	HRR60 ≤ 12 bpm (N = 45)	HRR60 > 12 bpm (N = 99)	*P* value	HRR120 < 22 bpm (N = 35)	HRR120 ≥ 22 bpm (N = 109)	*P* value
Myocardial infarction time to echocardiography and exercise test time interval (d)	36 ± 8	34 ± 6	0.280	36 ± 10	34 ± 5	0.255
Rest heart rate (bpm)	83 ± 17	81 ± 13	0.447	83 ± 17	81 ± 13	0.395
Maximal heart rate (bpm)	140 ± 19	145 ± 16	0.064	134 ± 19	147 ± 15	0.001
Systolic blood pressure (mm Hg)	123 ± 16	119 ± 16	0.175	124 ± 16	119 ± 16	0.085
Diastolic blood pressure (mm Hg)	76 ± 7	76 ± 8	0.982	76 ± 7	76 ± 8	0.992
Exercise time (min)	6.2 ± 2.0	7.8 ± 2.1	< 0.001	5.8 ± 2.0	7.8 ± 2.0	< 0.001
Metabolic equivalents	7.5 ± 2.3	9.4 ± 2.1	< 0.001	7.3 ± 2.1	9.3 ± 2.2	< 0.001
HRR60	7 (3–10)	19 (15–23)	< 0.001	7 (3–10)	18 (15–23)	< 0.001
HRR120	19 (12–24)	34 (29–39)	< 0.001	17 (11–19)	34 (27–38)	< 0.001

HRR60; Heart rate recovery at 60 s after exercise termination (Maximal heart rate – Heart rate at 60 s after exercise termination), HRR120; Heart rate recovery at 120 s after exercise termination (Maximal heart rate – Heart rate at 120 s after exercise termination)

**Table 4:** Mean and standard deviation of the 2D speckle-tracking echocardiography-derived parameters of the longitudinal deformation of the left atrial myocardium in the studied groups

Variables	HRR60 ≤ 12 bpm (N = 45)	HRR60 > 12 bpm (N = 99)	*P* value	HRR120 < 22 bpm (N = 35)	HRR120 ≥ 22 bpm (N = 109)	*P* value
LASr (%)	27.9 ± 7.6	30.4 ± 6.3	0.038	25.2 ± 5.2	31.0 ± 6.6	< 0.001
LAScd (%)	9.5 (7.2–13.2)	11.5 (9.4–13.9)	0.024	8.7 ± 2.7	12.4 ± 4.4	< 0.001
LASct (%)	17.2 ± 4.9	18.5 ± 4.5	0.139	16.5 ± 4.7	18.6 ± 4.5	0.022
pLASRr (s^− 1^)	2.7 ± 0.6	3.0 ± 0.7	0.024	2.6 ± 0.6	3.0 ± 0.7	0.002
pLASRcd (s^− 1^)	2.3 ± 0.8	2.8 ± 0.9	0.001	2.0 ± 0.6	2.9 ± 0.9	< 0.001
pLASRct (s^− 1^)	3.8 ± 1.3	4.3 ± 1.3	0.072	3.7 ± 1.3	4.3 ± 1.3	0.030

LAScd, Left atrial longitudinal strain during the conduit phase; LASct, Left atrial longitudinal strain during the contraction phase; LASr, Left atrial longitudinal strain during the reservoir phase; pLASRcd, Peak left atrial longitudinal strain rate during the conduit phase; pLASRct, Peak left atrial longitudinal strain rate during the contraction phase; pLASRr, Peak Left atrial longitudinal strain rate during the reservoir phase

### HRR60 ≤ 12 bpm vs. HRR60 > 12 bpm

Patients with abnormal HRR60 were older than those with normal HRR60 (*P* = 0.001). The prevalence of hypertension and diabetes was higher in patients with abnormal HRR60 (*P* = 0.016 and *P* = 0.004, respectively) (Table 1). In patients with abnormal HRR60, the E/e′ ratio (*P* < 0.001) and the prevalence of grades II and III LV diastolic dysfunction were higher (*P* = 0.022), whereas LVEF was lower (*P* = 0.018). The pre-P LA volume index and the minimal LA volume index were higher in patients with abnormal HRR60 (*P* = 0.044 and *P* = 0.034, respectively) (Table 2). The mean duration of the exercise test was shorter among patients with abnormal HRR60 than among patients with normal HRR60 (*P* < 0.001) (Table 3). LASr (30.4%±6.3 vs. 27.9%±7.6; *P* = 0.038), pLASRr (3.0 s^− 1^±0.7 vs. 2.7 s^− 1^ ±0.6; *P* = 0.024), LAScd (11.5% [9.4–13.9] vs. 9.5% [7.2–13.2]; *P* = 0.024), and pLASRcd (2.8 s^− 1^±0.9 vs. 2.3 s^− 1^±0.8; *P* = 0.001) were lower in patients with abnormal HRR60. The interval between MI occurrence and post-discharge echocardiography and the exercise test was not significantly different between the 2 groups.

The multivariable regression analysis after adjustments for potential confounders, consisting of age, hypertension, diabetes, LVEF, the E/e′ ratio, and the exercise duration, demonstrated that the differences between the 2 groups regarding LASr, pLASRr, logLAScd, and pLASRcd were lost.

### HRR120 < 22 bpm vs. HRR120 ≥ 22 bpm

Patients with normal HRR120 were younger than those with abnormal HRR120 (*P* < 0.001). The prevalence of diabetes was lower in patients with normal HRR120 (*P* = 0.013) (Table 1). In patients with normal HRR120, the E/e′ ratio was lower (*P* = 0.004). The maximal LA volume index, the pre-P LA volume index, and the minimal LA volume index were lower in patients with normal HRR120 (all *P*s < 0.001). All the volumetric parameters of LA phasic functions were higher in patients with normal HRR120, except for the booster active emptying percentage of total emptying, which was less in patients with normal HRR120 (all *P*s < 0.05) (Table 2). The mean duration of the exercise test was longer among patients with normal HRR120 than patients with abnormal HRR120, with the former group having a maximal heart rate at exercise time (*P* < 0.001 and *P* = 0.001, respectively) (Table 3). All the longitudinal deformation markers of the LA myocardium were higher in patients with normal HRR120 (all *P*s < 0.05) (Table 4). The time interval between MI occurrence and post-discharge echocardiography and the exercise test was not significantly different between the 2 groups.

The multivariable regression analysis after adjustments for potential confounders, consisting of age, diabetes, LVEF, the E/e′ ratio, the exercise duration, and the maximal LA volume index, indicated that the difference between the 2 groups remained regarding LAScd (*β* = 0.193; *P* = 0.017) and pLAScd (*β* = 0.198; *P* = 0.019).

The results concerning inter and intraobserver variabilities are presented in Table 5.

**Table 5:** Intra and interobserver variabilities for the 2D speckle-tracking echocardiography-derived parameters of left atrial myocardial function

Variables	Intraobserver		Interobserver	
	ICC	95% limit of agreement	ICC	95% limit of agreement
LASr (%)	0.982	0.947–0.993	0.915	0.803–0.964
LAScd (%)	0.840	0.632–0.930	0.817	0.556–0.923
LASct (%)	0.937	0.530–0.981	0.824	0.600–0.923
pLASRr (s^− 1^)	0.988	0.939–0.996	0.952	0.891–0.979
pLASRcd (s^− 1^)	0.995	0.987–0.998	0.992	0.982–0.997
pLASRct (s^− 1^)	0.994	0.985–0.997	0.953	0.891–0.980

ICC, Intraclass correlation coefficient; LAScd, Left atrial longitudinal strain during the conduit phase; LASct, Left atrial longitudinal strain during the contraction phase; LASr, Left atrial longitudinal strain during the reservoir phase; pLASRcd, Peak left atrial longitudinal strain rate during the conduit phase; pLASRct, Peak left atrial longitudinal strain rate during the contraction phase; pLASRr, Peak left atrial longitudinal strain rate during the reservoir phase

## Discussion

In the present study, for the first time, we employed 2DSTE to evaluate LA phasic functions in patients with recent acute MI with normal and abnormal HRR at 60 and 120 s. We found that the LA reservoir function markers, namely LASr and pLASRr, and the LA conduit function markers, namely LAScd and pLASRcd, were low in patients with abnormal HRR60 in comparison with patients with normal HRR60. Nevertheless, these differences were lost after adjustments for potential confounders. Additionally, there was a decline in the LA reservoir function markers, namely LASr and pLASRr, the LA conduit function markers, namely LAScd and pLASRcd, and the LA contraction function markers, namely LASct and LASRct, in patients with abnormal HRR120 compared with patients with normal HRR120. However, after adjustments for potential confounders, only the difference between the 2 groups remained statistically significant in terms of the LA conduit function markers.

Tadic et al. [20] in 2014 assessed LA phasic functions and HRV indices in patients with normal LVEF divided into groups with and without hypertension. Their results indicated a correlation between LASr and several indices of HRV, including the indices of the sympathetic and parasympathetic systems. They excluded patients aged > 60 years and those with diabetes or coronary artery disease. In contrast, our patients were affected by MI, and we did not exclude patients with diabetes and reduced LVEF.

Tadic et al. [21] in 2017 investigated LA phasic functions and HRV parameters in subjects with normal LVEF divided into groups with and without diabetes and revealed that a parameter of the parasympathetic system was correlated with LASr. Our study population, in contrast to theirs, included patients with reduced LVEF, hypertension, and a recent history of MI.

Vukomanovic et al. [22] in 2020 evaluated LA phasic functions and exercise capacity in patients with normal LVEF and without a history of coronary artery disease divided into groups with and without diabetes. Their results demonstrated a correlation between LASr and HHR60. In comparison with their study, we included patients with a history of recent MI, hypertension, and reduced LVEF. This group of researchers, in another study, found a correlation between endocardial right ventricular strain and HRR60 in a study population similar to that in their previous study. [33]

Exercise induces sympathetic system activation, accompanied by the withdrawal of the parasympathetic system. The recovery phase includes an initial fast phase, mainly dependent on the reactivation of the parasympathetic system, and a late slow phase, principally dependent on sympathetic withdrawal. [34] HHR60 and HHR120 are indices of these phases, respectively. [13] Our study results may indicate that the LA conduit function has a higher correlation with the late slow phase of HRR, suggesting sluggish sympathetic inactivation after adjustments for exercise duration. This conclusion is in alignment with the findings of a previous study indicating the superiority of HHR120 over HHR60 as a predictor of mortality. [35] Furthermore, it has been previously demonstrated that the LA conduit function is correlated with functional capacity in patients after MI, [12] patients with heart failure, [36, 37] and subjects with normal structural heart. [11] However, in the current study, we revealed that HRR120, independent of functional capacity, was correlated with the LA conduit function, which may point to the significance of autonomic dysfunction beyond functional capacity as an indicator of the LA conduit function.

Inflammation could lead to autonomic dysfunction [38] and LA phasic function in the presence of impaired systemic inflammation. [39] MI leads to the creation of an inflammatory milieu, increased cytokine levels, and increased oxidative stress [40], which can result in autonomic dysfunction and LA phasic dysfunction. Moreover, MI can lead to sympathetic overactivity, which persists for several months and is accompanied by decreased HRR. [2, 34] MI can also result in LV systolic dysfunction, accompanied by increased sympathetic activity and decreased parasympathetic activity. The increased sympathetic activity can be detrimental to myocardial function. [34] Further, MI can lead to LV diastolic dysfunction, which is one of the determinants of LA phasic functions, including the conduit function. [41] In the presence of MI, the activation of the renin-angiotensin-aldosterone axis occurs, with the increased aldosterone level associated with the LA conduit function. [42] There are possible pathophysiological mechanisms that can explain our findings.

Some studies have indicated the prognostic role and clinical significance of the LA conduit function. Diminished LA conduit function is present in patients with recent MI. The impairment of the LA conduit function assessed by cardiac magnetic resonance imaging is considered a predictor of major adverse cardiac events in patients with MI. [6, 43] In a prior study on patients with dilated cardiomyopathy, the LA conduit function was a predictor of a composite of sudden or cardiac death, heart failure hospitalization, and life-threatening arrhythmias. [44] In another investigation, the LA conduit function was a predictor of all-cause mortality and heart transplantation, and a composite of all-cause mortality, heart transplantation, heart failure readmission, and aborted sudden cardiac death. [45] In older adult subjects, the LA conduit function can augment accuracy in the prediction of death and heart failure with reduced or preserved EFs. [46] In a prior investigation, impaired LA conduit function was associated with the occurrence of AF in patients with MI after about 5 years of follow-up. [47] In addition, the LA conduit function can predict AF recurrence after electrical cardioversion and catheter ablation. [48, 49] On the other hand, autonomic dysfunction is associated with ventricular and atrial arrhythmias, such as AF, [34, 50] and increased risks of all-cause and cardiovascular mortality. [51]

In the prevention of cardiac autonomic dysfunction, it seems that general public health recommendations, such as weight reduction, healthy diets, increased physical activity, better behavioral stress management, and better control of cardiovascular risk factors (e.g., hypertension, diabetes, and dyslipidemia) should be considered. Moreover, some drugs used in treating patients after MI, such as β-blockers, angiotensin-converting enzyme inhibitors, and angiotensin receptor blockers, have been suggested in some studies. [51] In some cases, cardiac sympathetic denervation, renal denervation, and parasympathetic stimulation can be drawn upon. [50] Subjects with some of these risk factors for cardiac autonomic dysfunction have lower LA conduit function. [52] Hence, it seems that more appropriate treatment of factors that can lead to cardiac autonomic dysfunction may contribute to better LA conduit function. [53]

From a clinical perspective, our study indicates that a post-MI exercise test, which does not require sophisticated software and trained personnel, may be useful in providing information regarding the LA conduit function, functional capacity, and autonomic function. It, therefore, seems that the exercise test may be a time and cost-saving procedure, especially in the absence of advanced technology for the anticipation of impaired LA conduit function.

### Study Limitations

Our cross-sectional study indicated a correlation between 2 variables and did not indicate a causal relation. The single-center small-size study was another shortcoming, as a result of which some 2DSTE-derived markers of LA phasic functions that were significantly different between the 2 groups could have been rendered nonsignificant after adjustments for possible confounders. However, we could not evaluate LA functions by cardiac magnetic resonance imaging or 3D echocardiography. In addition, we used software initially designed for the assessment of LV deformation markers.

## Conclusions

In our patients with ST elevation MI treated by primary percutaneous intervention, HRR120 was correlated with the 2DSTE-derived markers of the LA conduit function after adjustments for possible confounding factors, including the exercise duration.

## Data Availability

The data sets analyzed in the current study are available from the corresponding author upon reasonable request.
